# Heparanase upregulation from adipocyte associates with inflammation and endothelial injury in diabetic condition

**DOI:** 10.1186/s12919-019-0181-x

**Published:** 2019-12-16

**Authors:** Nur Arfian, Wiwit Ananda Wahyu Setyaningsih, Muhammad Mansyur Romi, Dwi Cahyani Ratna Sari

**Affiliations:** Department of Anatomy, Faculty of Medicine, Public Health, and Nursing, Universitas Gadjah Mada, Yogyakarta, Indonesia

**Keywords:** Diabetes mellitus, Heparanase, MCP-1, Inflammation, Endothelial injury

## Abstract

**Background:**

Diabetes Mellitus (DM) is one of the metabolic diseases which leads to fatty tissue injury, and consequently inducing lipotoxicity and cellular senescence. This condition contributes to endothelial dysfunction with chronic inflammation and organ damage. Heparanase which has a role in disrupting endothelial surface layer (glycocalyx) may promote endothelial Nitric oxide synthase (eNOS) reduction and inflammation. However, its relationship with DM and organ injury has not been fully elucidated yet. This study aimed to determine how heparanase from fatty tissue may contribute to endothelial dysfunction and inflammation in patients with hyperglycemia and in a hyperglycemia model in rats.

**Methods:**

This population study with a cross-sectional design was conducted with 28 subjects without diagnosis and medication of DM. Fasting blood glucose levels, lipid profile, heparanase protein, MCP-1 protein and HbA1c were quantified. In vivo study was performed with a diabetic model in Wistar rats induced with streptozotocin 60 mg/kg body weight by single intraperitoneal injection. Rats were euthanized after 1 month (DM1 group, *n* = 6), 2 months (DM2 group, *n* = 6) and 4 months (DM4 group, *n* = 6). White Adipose Tissue (WAT) was harvested from visceral fat. Real Time and Reverse Transcriptase-PCR (RT-PCR) was done to quantify expressions of heparanase, MCP-1, eNOS, IL-6 and p-16 (senescence). Immunostaining was performed to localize MCP-1 and macrophage (CD68). Western blot tests were used to examine eNOS, MCP-1 and heparanase protein expression.

**Results:**

This study revealed associations between blood glucose levels with higher HbA1c, LDL, cholesterol, heparanase and MCP-1. The in vivo study also revealed lipid levels as the source of Heparanase and MCP-1 mRNA and protein expressions. This finding was associated with inflammation, cellular senescence and macrophage infiltration in fat tissue based on immunostaining and qRT-PCR analysis. RT-PCR revealed significantly lower expression of eNOS and higher expression of IL-6 in DM groups compared to the control group.

**Conclusion:**

Heparanase upregulation in fat tissue was associated with endothelial injury and inflammation in hyperglycemia conditions.

## Background

Type 2 Diabetes Mellitus (T2DM) is a metabolic disorder that causes increased morbidity and mortality every year. Worldwide, the number of patients with T2DM is expected to double by 2030 [1]. Indonesia was recognized among the ten highest global prevalence of DM in 2000, and is likely to maintain that status until 2030 [2]. Obesity is one of the main risk factors that leads to the T2DM development. Obesity induces leptin upregulation, adiponectin downregulation and resistin upregulation, which consequently leads to unhealthy metabolism changes. Those alterations include adipocyte hypertrophy which induces increases in free fatty acid levels. All of these phenomena are involved in lipotoxicity [3].

Lipotoxicity induces glucose homeostasis alterations due to failure of insulin signaling [4, 5]. Increased changes in metabolism in obesity and lipotoxicity bring upregulation of proinflammatory cytokines, chemokines and growth factors. These conditions may lead to macrophage infiltration and production of inducible-nitrite oxide synthase (iNOS), and oxidative stress (free radicals) [4]. Furthermore, obesity causes hyperinsulinemia, as a consequence of insulin resistance and hyperglycemia. Hyperglycemia becomes the origin of metabolic and structural disruptions, including increased production of reactive oxygen species (ROS), advance-glycation end products (AGE), and disruption of renin-angiotensin system (RAS) regulation [6, 7].

Diabetes mellitus type 2 is an important contributor to the occurrence of microvascular injury and organ dysfunction. The mechanism is based on the presence of sterile chronic inflammation, and cellular senescence. Senescence cells produce senescence-associated secretory phenotype (SASP) factors, including chemokines, proteases, proinflammatory cytokines, growth factors, macrophage inflammatory proteins (MIPs), and granulocyte-macrophage colony-stimulating factors (GM-CSFs) [7, 8]. The SASP components, such as interleukin (IL)-6, IL-8, and MCP-1, are elevated in obese adults and adolescents and may contribute to proinflammatory conditions. Another SASP component, PAI-1 is known to increase in circulation and tissue, as in coronary arteries [7]. Fat cells (adipocytes) play an important role in the pathogenesis of DM. These cells undergo accelerated senescence and induce injury to other cells, especially endothelial cells in the micro blood vessels. This suggests that endothelial cells are the key organ that plays a role in the pathophysiology of diabetic complications [9]. Endothelial injury may lead to complication of DM, such as ischemic reperfusion injury conditions, such as stroke, acute myocardial infarction, peripheral arterial diseases and kidney ischemic/reperfusion injury (IRI).

Endothelial injury can be induced by perfusion disturbance, such as in kidney ischemic/reperfusion injury [10]. Ischemic and hypoxic conditions in other organs, such as heart and liver also lead to endothelial damage with a disruption of glycocalyx [11–14]. In the kidney diseases, glycocalyx disruption and shedding have been already known occur in inflammation and ischemic condition [15, 16], albuminuria and microvascular permeability [17] and adriamycin nephropathy [18]. Glycocalyx is composed by proteoglycan components consisting of heparan sulphate and chondroitin sulphate. Among proteoglycan, heparan sulphate (HS) is the most common glycosaminoglycan (GAG) in the endothelial cell (EC), constitute 40–50% of EC glycocalyx [16]. HS loss is also induced by heparanase production, an endoglycosidase that degrades HS [19]. Our previous study revealed upregulation of heparanase in kidney ischemic reperfusion injury model, which associated with reduction of eNOS protein expression and degradation of endothelial surface layer/glycocalyx. Heparanase upregulation with eNOS reduction and Endothelin-1 elevation also occurred in hypoxic endothelial cells culture. This associated with upregulation of Intercellular Adhesion Molecule-1 (ICAM-1) [20]. Heparanase have been reported play roles in cancer metastasis and invasion through interstitial remodeling [21–24]. It also plays role in renal diseases induced by hyperglycemia and diabetes [25, 26]. So far, there is no report about the expression of heparanase in prediabetic to diabetic condition in correlation with endothelial injury, inflammation and lipid profiles. This study elucidate expression of heparanase in prediabetic and diabetic condition with obese population. We also confirmed heparanase expression in diabetic mellitus model in rats.

## Methods

### Subject characteristics

This research was a cross-sectional population study. Fasting blood samples were collected from an obese population involving 24 subjects, consisting of females with ages 41–89 years old and living in the rural area of Bantul, Yogyakarta. Inclusion criteria were: subjects without diagnosis of DM and no medication of DM and willing to participate in the study. Exclusion criteria were patients with chronic diseases, DM medication, and who refused to join the study. All subjects completed informed consent forms. The study was approved by the Medical and Health Research Ethics Committee of the Faculty of Medicine, Public Health and Nursing, Universitas Gadjah Mada, Yogyakarta, Indonesia. All of the subjects were examined for basic anthropometric data including: body weight, height, blood pressure, serum blood glucose and abdominal circumstance. Abdominal circumference (AC) was used to determine obese patients with AC > 80 cm.

### Study groups

Subjects were divided into 4 groups based on their Body Mass Index (BMI). Group K1 consisted of subjects with normal BMI and fasting blood glucose (FBG) less than 100 mg/dL (normal, *n* = 10), group K2 were subjects with AC > 80 and FBG less than 100 (normal, *n* = 10), group K3 were subjects with AC > 80 (obese) and FBG 100–125 mg/dL (prediabetic condition, *n* = 10), while K4 group included subjects with AC > 80 cm (obese) and FBG > 125 mg/dL (n = 10). Nearly 100 people were examined in a countryside population for screening of diabetes and obesity. Subjects diagnosed as obese (AC > 80) were included in the study. Subject who have already diagnosed diabetes mellitus and got medication were excluded from the study. After explanation of the research objectives and informed consent, patients were checked for fasting blood analysis.

### Blood serum analysis

Fasting blood samples were taken from the subjects as much as 4.5 mL and kept in vacutainer with EDTA for anti-coagulant. Serum was made using centrifugation with 10,000 rpm for 10 min in 4 °C temperature. Serum was kept in -80 °C. Lipid profiles (LDL, Triglycerides, HDL, and Cholesterol) were quantified in a clinical laboratory using standard methods.

### Heparanase, monocyte chemoattractant protein-1 and HbA1c quantification

Serum was also used for quantification of heparanase (Finetest, EH1020), Monocyte chemoattractant protein-1 (MCP-1) (Finetest, EH022) using ELISA kit. All of the procedures were based on instruction manual of the kit. Level of HbA1c was also examined in clinical laboratory using standard method.

### Animal model of diabetes mellitus (DM)

A total of 24 male Sprague Dawley mice age 3–4 months were used in this study. Rats were placed in cages with a light-dark cycle of 12 h. DM model was induced with single intraperitoneal injection of Streptozotocin (Nacalai, 32,238–91) at 60 mg/kg body weight. Blood glucose levels were quantified on day 5 after injection to examine the success of the model. DM was defined if the blood glucose level was higher than 200 mg/dL. Rats were divided based on the time of euthanasia, 1 month (DM1 group, *n* = 7), 2 months (DM2 groups, n = 7), 4 months (DM4 group, n = 7). The control group was injected with NaCl 0.9% for single dose, then euthanized after 4 months.

For euthanasia methods, rats were anaesthetized using ketamine at a dose of 60-100 mg/kg BW intramuscularly (i.m.). Abdomen and thorax were opened after deep anaesthesia, and the left ventricle was perfused with NaCl 0.9%. Visceral fat tissues were harvested from intraperitoneal and perirenal areas, then kept in Normal Buffer Formalin for paraffin making and RNA preservation solution for RNA extraction.

### RNA extraction, cDNA synthesis and reverse transcriptase-polymerase chain reaction (RT-PCR)

The RNA from fat tissues were extracted using Genezol solution (GENEzol™, Cat No. GZR100) based on the manufacturer’s protocol. RNA concentrations were quantified using a nanodrop. The synthesis of RNA to cDNA was done using ReverTra Ace® (Toyobo, Cat. No. TRT-101), deoxyribonucleotide triphosphate (dNTP) (Takara, Cat. No. 4030), and primary random (TAKARA, Cat No. 3801).

Reverse Transcriptase-PCR (RT-PCR) was performed for these following genes with specific primers: heparanase (forward: 5′-CGAACGTCTATCACCCAAGGT-3′).; reverse: 5′-AGAACCGAAAGGCTTCAGCA-3′).), eNOS (forward: 5-CCGGCGCTACGAAGAATG-3′).; reverse: 5′-AGTGCCACGGATGGAAATT-3′).), Interleukin-6 (forward: 5′-TTGGATGGTCTTGGTCCTTAGCC-3′).; reverse: 5′-TCCTACCCCAACTTCCAATGCTC-3′).) and a housekeeping gene, ß-actin (forward 5′- GCAGATGTGGATCAGCAAGC-3′ and reverse 5′-GGTGTAAAACGCAGCTCAGTAA-3′). The PCR was performed using the following condition: initial denaturation 94 °C for 2 min, the following steps were repeated for 35 cycles (denaturation 94 °C for 20 s, annealing 60 °C for 20, elongation 72 °C for 1 min), and last extension 72 °C for 10 min.

For RT-PCR, we used Taq Master Mix (GoTaq®Green Master Mix, Cat No. M7122). PCR products were analyzed on 2% agarose gel with DNA ladder (Bioron, Germany, Cat No. 306009). Gene expression was quantified with densitometric analysis using ImageJ software and GAPDH was used to normalize expression.

### Immunohistochemical (IHC) staining of MCP-1 and CD68 from visceral adipose tissue

The visceral adipose tissue from intraperitoneal were made for paraffin, the slides were deparaffinized, then heated incitrate buffer, incubated with 3% H2O2 in PBS for endogenous peroxidase inhibition and incubated with blocking solution. Furthermore, the slides were incubated with anti-CD68 (1/100 dilution, Abcam, ab955) and MCP-1 antibody (1/100 dilution, Abcam, ab25124) at 4 °C overnight. After slides were incubated with species-specific secondary antibodies for 1 h at room temperature, slides were incubated with avidin-HRP and counter stained with DAB (Biocare, STUHRP700H) complete antigen detection was used avidin-biotinylated complex-horseradish peroxidase before DAB staining. Slide were conterstained with haematoxylin.

### Protein extraction and Western blot

Protein from adipose tissue was extracted using the Pro-Prep™ (Intron Biotechnology; Cat. No. 17081) from white adipose tissue based on manufacturer instructions. Thirty milligrams of adipose tissues were homogenized with 600 μL of Pro-Prep™ solution. The homogenates were centrifuged at 12,000 rpm at 4 °C for 20 min. The supernatants were stored in safe lock tubes at -80 °C until they were assayed. A total of 40 g of protein was separated onto 10% SDS-PAGE, and transferred to a polyvinylidene fluoride membrane (PVDF) and incubated with anti-heparanase (anti-rabbit, 1:500 dilution), Anti-eNOS (anti-rabbit, 1:300) and anti-MCP-1 (anti-rabbit, 1:1000 dilution). A total of 5% skim milk in TBST was used for blocking followed by incubation with the appropriate secondary antibody. Proteins were visualized using ECL Prime Western Blotting Detection Reagents (GE Healthcare, RPN2232). Blots were photographed with a Geldoc machine (Geldoc Syngene Gbox Seri Chemi xrq).

## Results

### Obese with DM condition associated with higher cholesterol and triglycerides

**Fig. 1:**
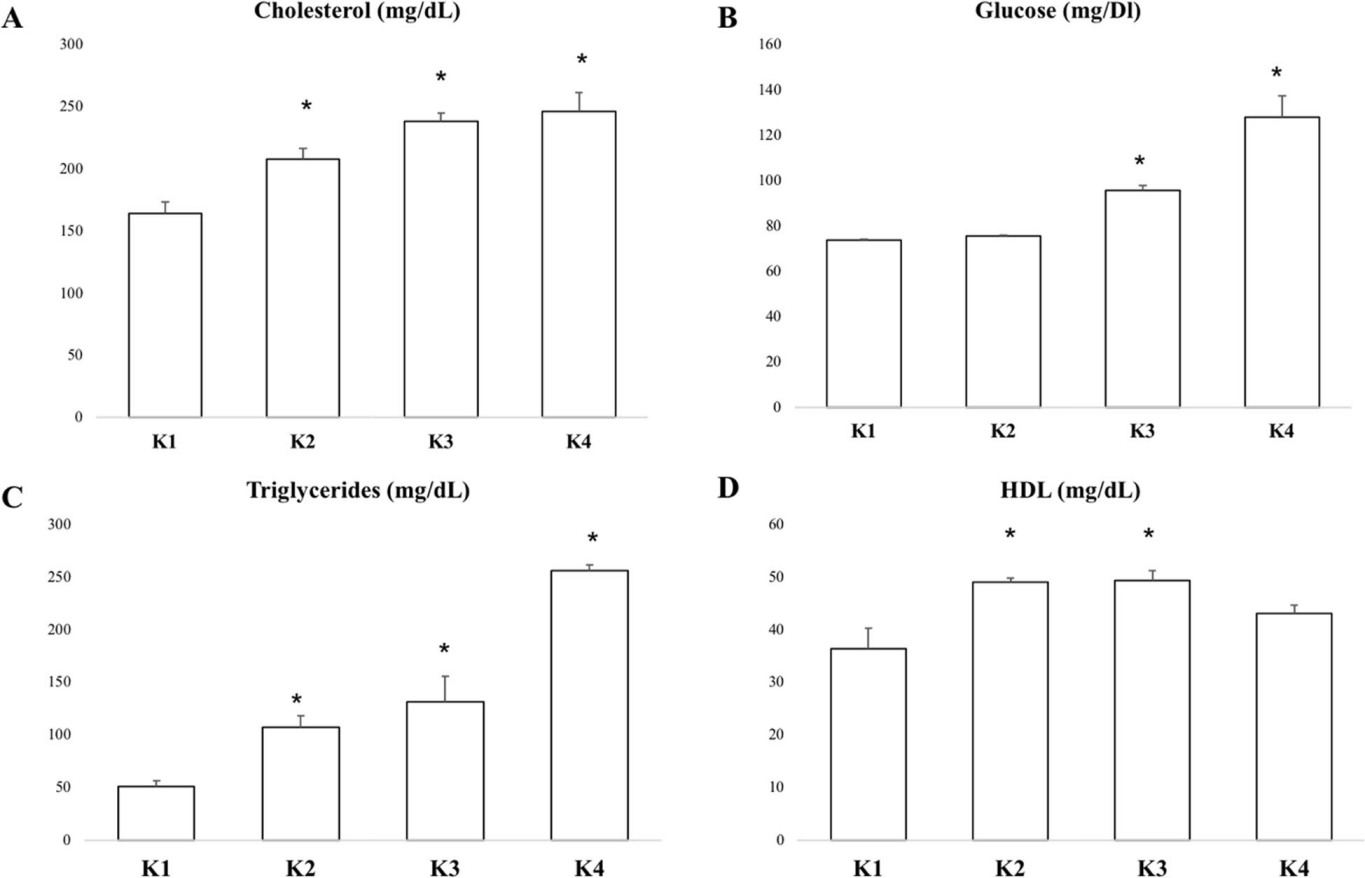
Mean of cholesterol (**a**), fasting glucose (**b**), triglyceride (**c**) and HDL (**d**) levels in each group. Higher glucose levels might be associated with higher cholesterol and triglyceride levels

The K1 group represented normal conditions with normal BMI and normal glucose levels. Meanwhile, K2, K3 and K4 groups represented obese groups with different levels of glucose. Obese subjects in the K2, K3 and K4 groups had significantly higher cholesterol and triglyceride levels compared to K1, as the representative of normal conditions. However, K2 and K3 groups had significantly higher HDL levels compared to K1. The K4 group had significantly lower HDL level compared to the K2 and K3 groups. This result suggests higher glucose levels might be associated with lower HDL levels (Fig. 1).

### Higher glucose levels associated with increased endothelial dysfunction, heparanase expression and inflammation

**Fig. 2:**
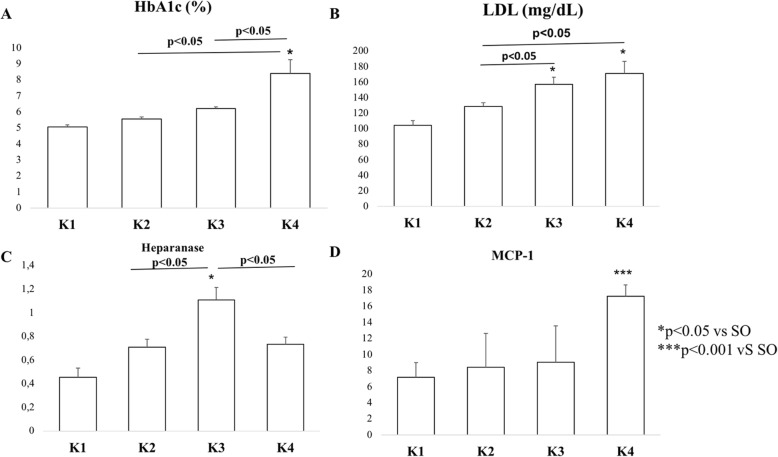
**a** Mean of HbA1c level in each group showed highest HbA1c level in K4 group. **b** LDL level demonstrated the highest LDL level in K4 group. **c** Heparanase protein levels with ELISA in each group. **d** MCP-1 protein level with ELISA revealed the highest level in K4 group

Next, we examined HbA1c as marker for glycation of endothelial cells, especially in diabetic condition for showing endothelial function and predicting arterial stiffness and endothelial dysfunction [27]. HbA1c concentration quantification revealed that K4 group had the highest HbA1c and significant higher compared to K1, K2 and K3 group. K4 group had mean of HbA1c more than 6 as cut point for endothelial injury. LDL level showed K3 and K4 groups had significant higher LDL level compared to K1 group, furthermore K4 group represented the highest LDL level. K4 group also had significant LDL level compared to K2 and K3 groups. It seemed that higher glucose level also associated with higher LDL level (Fig. 2b).

ELISA quantification of heparanase protein showed significantly higher levels in the K2, K3 and K4 groups compared to the K1 group. Interestingly, K3 group which represented prediabetic conditions had the highest heparanase protein levels among the groups. There were also significantly higher levels of heparanase protein in the K3 group compared to the K2 and K4 groups. Meanwhile, the MCP-1 protein level measurements showed the highest MCP-1 protein level in the K4 group, which was significantly different compared to the K1, K2 and K3 groups. There were no significant differences between groups K1, K2 and K3 (Fig. 2c-d).

### In vivo experiment showed adipocyte tissue as the source of heparanase and MCP-1

**Fig. 3:**
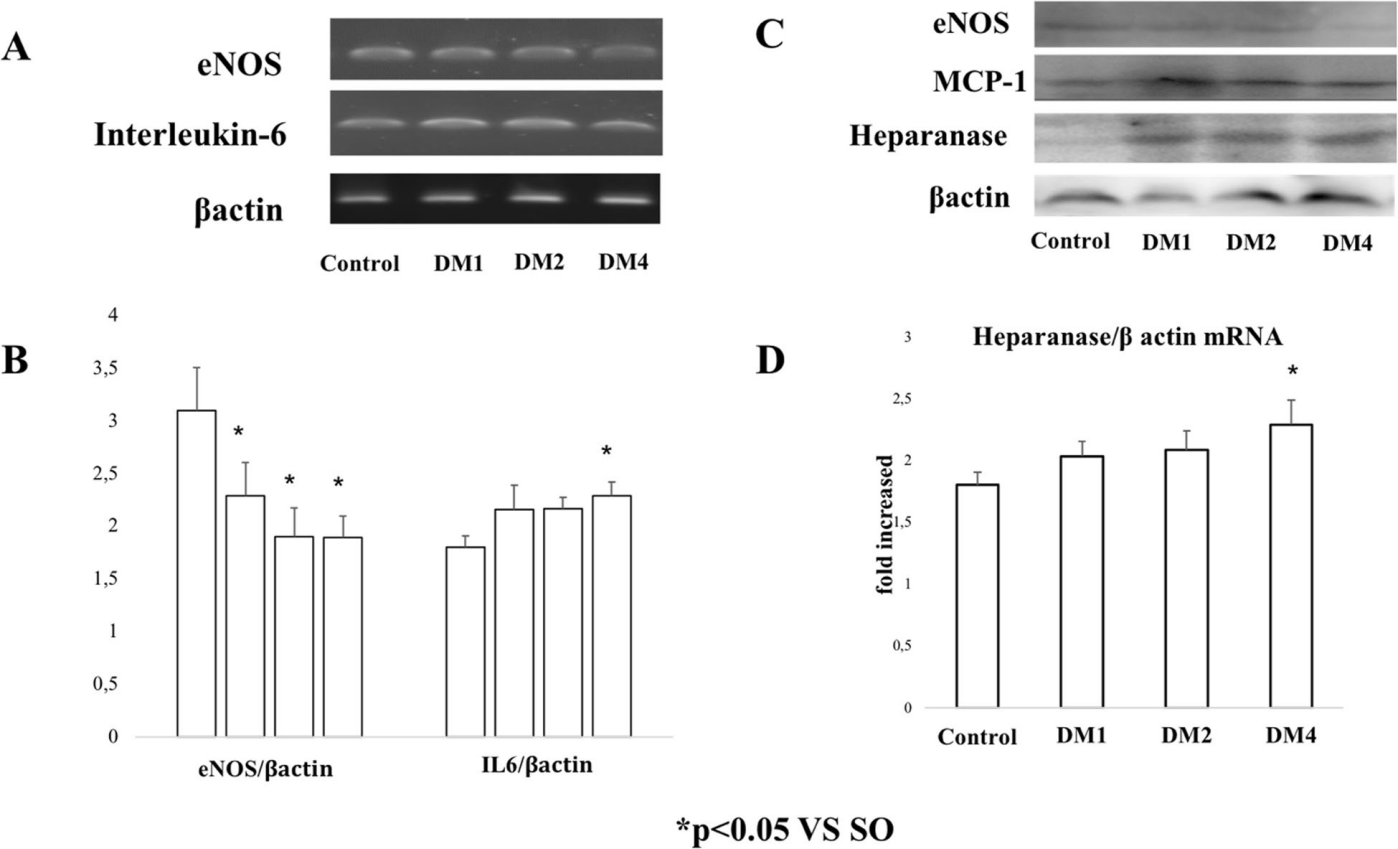
**a**-**b** RT-PCR analysis of eNOS (endothelial dysfunction marker) and IL-6 (inflammation marker). **c** Representative result of eNOS, MCP-1 and heparanase protein levels using Western blot methods. **d** qRT-PCR analysis of heparanase

In vivo experiment was conducted to confirm the source of heparanase and inflammation in the hyperglycemic conditions, which in groups DM1, DM2 and DM4 were demonstrated by significantly lower eNOS mRNA expressions compared to the SO group (Fig. 3 a-b). It was also associated with significantly lower IL-6 mRNA expressions as a biomarker of inflammation in the DM4 group, although the results showed that the IL-6 expression was lower in the hyperglycemic groups. However, statistical analysis revealed only the DM4 group had significant differences compared to the SO group.

Real Time-PCR (qRT-PCR) also revealed significantly higher expression of heparanase mRNA expression in adipose tissue of the DM4 group compared to the SO group. DM4 had the highest heparanase mRNA expression and had significantly higher expression compared to the DM1 and DM2 groups. Western blot analysis was done for representative and confirmation of heparanase, eNOS and MCP-1 expressions. It showed that the hyperglycemic conditions were associated with higher expression of heparanase and MCP-1 in adipocyte tissue. Interestingly, the lower eNOS expression was clearly demonstrated in the DM4 group.

### Immunostaining of MCP-1 and macrophage in adipocyte tissue

**Fig. 4:**
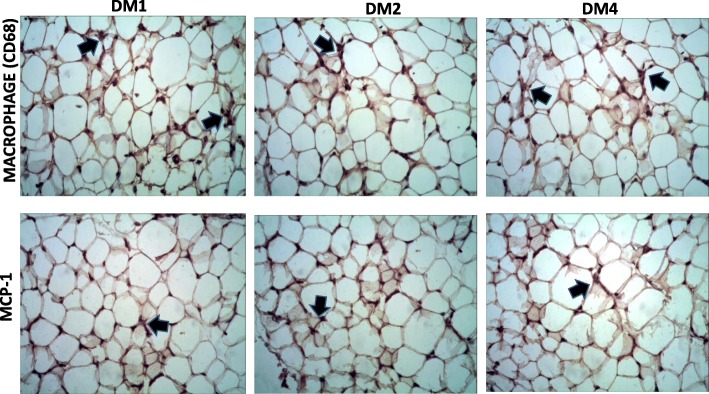
Immunostaining of CD68 and MCP-1 in adipose tissue (black arrows). The black arrows show the positive cells

Immunostaining of inflammation markers using MCP-1 and CD68 (macrophage) showed positive staining in adipocyte tissue in the hyperglycemic groups. CD68 as a macrophage marker had positive signaling in interstitial areas of the tissue which suggests macrophage infiltration might be induced by hyperglycemic conditions (Fig. 4).

## Discussion

This study reveals inflammation with endothelial injury occurs more in populations with obesity and high glucose levels. This condition may be associated with deterioration of fatty lipids with increasing LDL, triglycerides and cholesterol levels. Remarkably, Heparanase, an enzyme that can cleave the Heparan sulphate (HS) proteoglycan begins to increase in prediabetic condition. Patients with diabetes mellitus (DM) are at higher risk for many diseases such as cardiovascular diseases, peripheral arterial diseases, cerebrovascular diseases and chronic kidney diseases. These diseases lead to end stage organ damage such as retinopathy, nephropathy, and neuropathy [6, 28].

Obesity may correlate with increased blood glucose levels and inflammation. Obesity stimulates extrication of proinflammatory cytokines and chemokines causing insulin resistance. The excesses of insulin resistance give numerous impacts such as an increase of free fatty acid (FFA) from adipocyte tissue to endothelial cells. The increase of FFA leads to the production of reactive oxygen species (ROS) which activates either AGE, PKC, or hexosamine (GlcNAc) pathways or nuclear factor kappa beta (NFκB). Various pathways induce production of inducible nitric oxide synthase (iNOS), which contributes to the insulin resistance. Proinflammatory cytokines and chemokines, especially MCP-1, expressed by adipocyte cells, endothelial cells, and monocyte induce an increase of macrophage numbers [29, 30].

Proinflammatory cytokine and chemokine-induced by obesity stimulates sterile chronic inflammation which augments cellular senescence [7, 31]. The combination of hyperglycemia, insulin resistance, and chronic inflammation induced endothelial dysfunction, moreover macrovasculopathy. Endothelial cells (EC) may become the most susceptible cell to be injured in this condition. Hayasi (2006) showed that there is an attenuation of eNOS protein and augmentation of reactive oxygen species (ROS) in Human Umbilical Vascular Endothelial cells (HUVEC) treated with high concentration of glucose for 24 h. This condition may associates with cellular senescence in HUVEC [32].

ELISA results showed that an increase of heparanase protein was associated with upregulation of MCP-1, an inflammatory marker and HbA1, an endothelial glycation marker. Some examination may be needed for further study to analyze the oral glucose tolerance test and endothelial dysfunction, such as flow-mediated dilatation for endothelial dysfunction condition. Although we found that the increasing of heparanase occurred in the prediabetic conditions (as represented with K3 group), and decreased slightly in diabetic and obese conditions (represented with K4 group). Previous study demonstrated an increase of heparanase levels in diabetes patients, which can be detected in urine and plasma of the patients [26, 33]. The hyperglycemic condition also induces heparanase expression in endothelial cells [33, 34]. In vitro studies also revealed that heparanase expression upregulates from ECs and human embryonic kidney (HEK) cells under high glucose stimulation [33, 34]. Our results also showed that the prediabetic condition in the K3 group had the highest heparanase protein level, then became lower in the K4 group, although the differentiation was significant and remained higher compared to the K1 group (normal/control population). This finding suggests that high glucose levels may stimulate upregulation of heparanase. Heparanase-mediated degradation of heparan sulphate affect the migration of inflammatory cells, including neutrophils, macrophages, dendritic, and mast cells, and destroy the cells [35]. Heparanase might be secreted form adipocyte based on our animal study, however we did not differentiate whether adipocyte itself or vascular cells of adipocytes that might secret heparanase. Describing the source of heparanase in adipocyte may give better understanding for further study. Previous study found glucose was the main stimulator of heparanase, especially in rat glomerular epithelial cells and human embryonic kidney (HEK) 293 cells, which was associated with loss of HS [36]. Insulin and high glucose in obese and insulin resistance conditions might induce the heparanase upregulation [33].

## Conclusion

We conclude that upregulation of heparanase in fat tissue was associated with endothelial injury and inflammation in hyperglycemia conditions.

## Data Availability

All data generated or analysed during this study are included in the submission. The raw data are available from the corresponding author on reasonable request.

## Abbreviation

AC: Abdominal circumferrence
AGE: Advance-glycation end products
BMI: Body mass index
CD68: Cluster of differentiation 68
DM: Diabetes mellitus
EC: Endothelial cell
EDTA: Ethylenediaminetetraacetic acid
eNOS: Endothelial nitrite oxide synthase
FBG: Fasting blood glucose
FFA: Free fatty acid
GAG: Glycosaminoglycan
GlcNAc: N-Acetylglucosamine
GM-CSF: Granulocyte-macrophage colony-stimulating factors
HbA1c: Glycated haemoglobin (A1c)
HEK: Human embryonic kidney
HS: Heparan sulphate
ICAM-1: Intercellular adhesion molecule-1
IL6: Interleukin-6
iNOS: Inducible nitrite oxide synthase
IRI: Ischemic reperfussion injury
LDL: Low density lipoprotein
MCP-1: Monocyte chemoattractant protein-1
MIP: Macrophage inflammatory proteins
NF *κ* B: Nuclear factor kappa beta
PAI-1: Plasminogen activated inhibitor-1
qRT-PCR: Quantitative real time polymerase chain reaction
RAS: Renin-angiotensin system
RNA: Ribonucleic acid
ROS: Reactive oxygen species
RT-PCR: Reverse transcriptase polymerase chain reaction
SASP: Senescence-associated secretory phenotype
SDS-PAGE: Sodium duodecyl sulphate-Poly acrylamide gel electrophoresis
TBST: Tris buffer saline with Tween20
